# 

**Published:** 1879-12

**Authors:** 


### CONTENTS:

ORIGINAL LECTURES:

I. A Clinical Lecture on Tubercular Leprosy. By James Nevins Hyde, M.D. .......... 561

ORIGINAL COMMUNICATIONS:

II. Some Further Thoughts Concerning the Origin and Spread of Yellow Fever, and the Means of preventing it. By N. S. Davis, M.D. .......... 574

III. Diffuse Multiple Capillary Fat Embolism of the Lungs and Brain as a Fatal Complication in Common Fractures; Illustrated by a Case. By Chr. Fenger, M.D. and J. H. Salisbury, M.D. .......... 587

IV. Anomalous Complications of Uterine Fibroids. By J. T. Everett, M.D. .......... 606

V. Traumatic Insanity in its Medico-legal Relations. By Daniel R. Brower, M.D. .......... 609

VI. Dental Hints for Country Medical Practitioners. By G. Newkirk, M.D. .......... 615

VII. Alcoholism; its Treatment from an Analysis of One Hundred and Twenty-five Cases. By E. C. Helm, M. D. .......... 623

CLINICAL REPORTS:

*Notes from Hospital Practice:*

VIII. Two Cases of Traumatic Tetanus... 627

SOCIETY REPORTS:

IX. Chicago Medical Society .......... 631

DOMESTIC CORRESPONDENCE:

X. New York Letter .......... 33

XI. Gelatine Suppositories .......... 636

REVIEWS AND BOOK NOTICES .......... 637

*Books and Pamphlets Received* .......... 655

SELECTIONS:

The Perimetric Dimension System; a General System of Measurement for Urethral, Uterine, Rectal and Other Instruments; and an adaptable Metric Gauge .......... 658

SUMMARY:

*Pathological Anatomy:*

False Hermaphrodism .......... 664

*Practical Medicine:*

Artificial Digestion .......... 666

*Surgery:*

Indications and Contra-Indications for Operations on Subjects Affected with Constitutional Diseases .......... 667

OBITUARY .......... 670

ITEMS .......... 626, 632, 636, 663, 669

ANNOUNCEMENTS FOR THE MONTH .......... 672

THE METRIC SYSTEM .......... xxi, xxiv advts.

Notice to Contributors .......... xx advts.

